# IDH1 Non-Canonical Mutations and Survival in Patients with Glioma

**DOI:** 10.3390/diagnostics11020342

**Published:** 2021-02-19

**Authors:** Enrico Franceschi, Dario De Biase, Vincenzo Di Nunno, Annalisa Pession, Alicia Tosoni, Lidia Gatto, Giovanni Tallini, Michela Visani, Raffaele Lodi, Stefania Bartolini, Alba Ariela Brandes

**Affiliations:** 1 Department of Oncology, AUSL Bologna, 40139 Bologna, Italy; dinunnovincenzo88@gmail.com (V.D.N.); a.tosoni@ausl.bologna.it (A.T.); lidia.gatto83@gmail.com (L.G.); stefania.bartolini@ausl.bologna.it (S.B.); alba.brandes@yahoo.it (A.A.B.); 2 Department of Pharmacy and Biotechnology (Dipartimento di Farmacia e Biotecnologie)-Molecular Diagnostic Unit, Azienda USL di Bologna, University of Bologna, 40126 Bologna, Italy; dario.debiase@unibo.it (D.D.B.); annalisa.pession@unibo.it (A.P.); 3 Department of Medicine (Dipartimento di Medicina Specialistica, Diagnostica e Sperimentale)-Molecular Diagnostic Unit, Azienda USL di Bologna, University of Bologna School of Medicine, 40126 Bologna, Italy; giovanni.tallini@unibo.it (G.T.); michela.visani@unibo.it (M.V.); 4 IRCCS Istituto delle Scienze Neurologiche di Bologna, 40139 Bologna, Italy; raffaele.lodi@isnb.it

**Keywords:** glioma, WHO grade II glioma, WHO grade III glioma, IDH1, prognostic factor

## Abstract

Background: Non-canonical mutations of the isocitrate dehydrogenase (IDH) genes have been described in about 20–25% and 5–12% of patients with WHO grade II and III gliomas, respectively. To date, the prognostic value of these rare mutations is still a topic of debate. Methods: We selected patients with WHO grade II and III gliomas and IDH1 mutations with available tissue samples for next-generation sequencing. The clinical outcomes and baseline behaviors of patients with canonical IDH1 R132H and non-canonical IDH1 mutations were compared. Results: We evaluated 433 patients harboring IDH1 mutations. Three hundred and ninety patients (90.1%) had a canonical IDH1 R132H mutation while 43 patients (9.9%) had a non-canonical IDH1 mutation. Compared to those with the IDH1 canonical mutation, patients with non-canonical mutations were younger (*p* < 0.001) and less frequently presented the 1p19q codeletion (*p* = 0.017). Multivariate analysis confirmed that the extension of surgery (*p* = 0.003), the presence of the 1p19q codeletion (*p* = 0.001), and the presence of a non-canonical mutation (*p* = 0.041) were variables correlated with improved overall survival. Conclusion: the presence of non-canonical IDH1 mutations could be associated with improved survival among patients with IDH1 mutated grade II–III glioma.

## 1. Introduction

Molecular assessment represents a milestone for the diagnosis of gliomas. In-depth genomic evaluations provide crucial details about the clinical aggressiveness of tumors and can be used to inform the prognosis of the disease [1,2,3,4,5,6,7,8,9,10].

The 2016 World Health Organization (WHO) classification of primary central nervous system (CNS) tumors establishes that glioma pathological diagnosis must not leave out molecular examination [1].

The integration of objective parameters for diagnosis, such as the assessment of molecular markers, is essential in order to homogenize pathological diagnoses and reduce inter-observer variability.

Evaluation of isocitrate dehydrogenase (IDH) gene alterations represents one of the most critical variables to consider, since survival is drastically prolonged for patients harbouring an IDH mutation [1,2,3,4,5].

The incidence of an IDH mutation is very high in patients with WHO grade II–III glioma (more than 80%) and secondary glioblastoma (GBM) (73%), while it is uncommon among patients with primary GBM (3.7%) [2,3,4,5]. Gain-of-function of the IDH gene results in increased intracellular levels of 2-hydroxyglutarate, which leads to alterations of DNA methylation [11]. The final biological effect is cellular dedifferentiation and growth promotion, mainly mediated by gene transcription deregulation. Clinically, IDH-mutated tumors present prolonged survival and an improved response to chemotherapy as compared to IDH-wild type gliomas [11]. Indeed, IDH-wild type gliomas often amplify epidermal growth factor receptor (EGFR), gain of chromosome 7 and loss of chromosome 10 and telomerase reverse transcriptase (TERT) promoter mutations [11,12]. These biological features make IDH-wild type tumors biologically and clinically more similar to glioblastoma [11,12].

The 1p19q codeletion represents the hallmark alteration for IDH-mutated oligodendroglioma and is associated with prolonged survival and better response to chemotherapy [11]. The exact biological mechanism by which this alteration affects cell proliferation and tumor development is still unclear. We know that 1p19q co-deletion is mutually exclusive with loss of the nuclear expression of the alpha thalassemia mental retardation syndrome x-linked (ATRX) gene [11].

The R132H (c.395 G > A, exon 4 codon 132) mutation of the IDH1 gene is the most common alteration [3].

The availability of techniques capable of performing fast and deep genome sequencing, has allowed us to identify novel and uncommon IDH gene mutations. The term “non-canonical mutations” involves all mutations other than R132H IDH1 alterations. These unusual mutations have been reported in about 20–25% and 5–12% of patients with grade II and III gliomas, respectively, but are extremely uncommon in patients with GBM [13,14,15,16,17,18].

In the present study, we aimed to assess the clinical outcomes achieved by patients with grade II–III gliomas harbouring non-canonical IDH1 mutations.

## 2. Materials and Methods

### 2.1. Patient Selection

Patients were identified by a review of the active electronic chart system in our institution. Only patients with updated follow up data, adequate tumor tissue samples available for sequencing analysis, and proven IDH mutations were selected.

**Figure 1:**
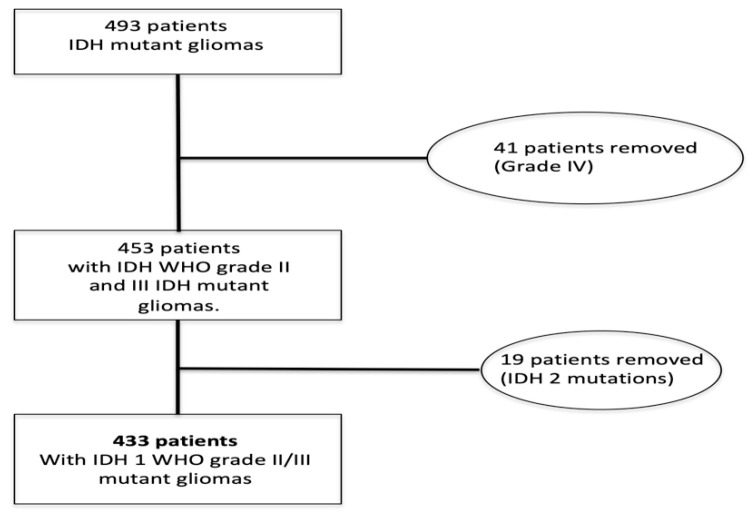
Patient selection. IDH: isocitrate dehydrogenase.

By adopting these inclusion criteria, we selected a population of 493 patients. After further revision, 60 patients were removed (41 had primary GBM, 19 patients had an IDH2 mutant glioma). Finally, we selected 433 patients with WHO grade II–III IDH1 mutant glioma from February 2008 to February 2020 (Figure 1).

The median follow up was 94.9 (95% CI, 85.9–104.0) months.

According to the 2016 WHO Classification, each tumor sample was reviewed as WHO grade II or III gliomas [1] and assessed by specific extended IDH gene sequencing. The study was approved by the Ethical Committee of Azienda Sanitaria Locale di Bologna (protocol number CE09113, Bologna, Italy). All information regarding the human material was managed using anonymous numerical codes, and all samples were handled in compliance with the Declaration of Helsinki.

### 2.2. IDH1 Analysis

DNA was extracted from FFPE (formalin fixed paraffin embedded) material (two to four 10 μm-thick sections deposited on glass slides, according to the amount of lesional tissue present in the paraffin block). The areas of interest were scraped under microscopic guidance using a sterile blade. DNA was extracted using the Quick Extract Kit (Epicentre, Madison, WI, USA).

IDH1 (exon 4, codons 96–138) analysis was performed using the following primers: IDH1-Exon4-Fw 5′-gAAACAATgTggAAATCACCA-3′ and IDH1-Exon4-Rc 5′-gAAACAAATgTggAAATCACCA-3′.

Amplicons were sequenced using the 454 GS-Junior NGS (Roche, Mannheim, Germany) according to previously described protocols [19,20].

Each IDH1 mutation that was different from the ‘’canonical’’ R132H (c.395 G > A, exon 4 codon 132) was defined as an IDH1 ‘’non-canonical mutation’’.

### 2.3. Statistical Analysis

Data were reported as median, range, and frequencies. Overall survival (OS) analyses were estimated through the Kaplan–Meier method and were analyzed by the means of a log-rank test and the forward stepwise multivariate Cox proportional hazards model. The hazard ratios (HRs) were computed together with their 95% confidence intervals (95%CIs).

Two tailed *p*-values < 0.05 were considered statistically significant. Statistical analysis was performed using SPSS version 13.0 (SPSS Inc., Chicago, IL, USA). The primary endpoint of the present study was the OS comparison between patients with IDH1 canonical and IDH1 non-canonical mutated grade II or III glioma.

## 3. Results

The studied population was composed of 433 patients with WHO grade II–III IDH1 mutant gliomas (Figure 1). Of these, 390 patients had a R132H IDH1 mutation (90.1%), while 43 patients had non-canonical IDH1 mutations (9.9%). The R132C and R132G mutations were the most frequent non-canonical IDH1 mutations detected, followed by the R132S and the R132L (Table 1).

The median age was 38 years (range 18–76 years). WHO grade II and grade III IDH1 mutant gliomas were diagnosed in 269 (62.1%) and 164 (37.9%) patients, respectively.

Compared to those with R132H IDH1 mutations, patients with non-canonical mutations were younger (*p* < 0.001), presented the 1p19q codeletion less frequently (*p* = 0.017), and more often received complete surgical resection (*p* = 0.039). All patients’ baseline characteristics are summarized in Table 2.

After a median follow-up of 94.9 months, 291 (67.2%) patients were still alive.

The median survival in the overall population was 149.7 months.

**Figure 2:**
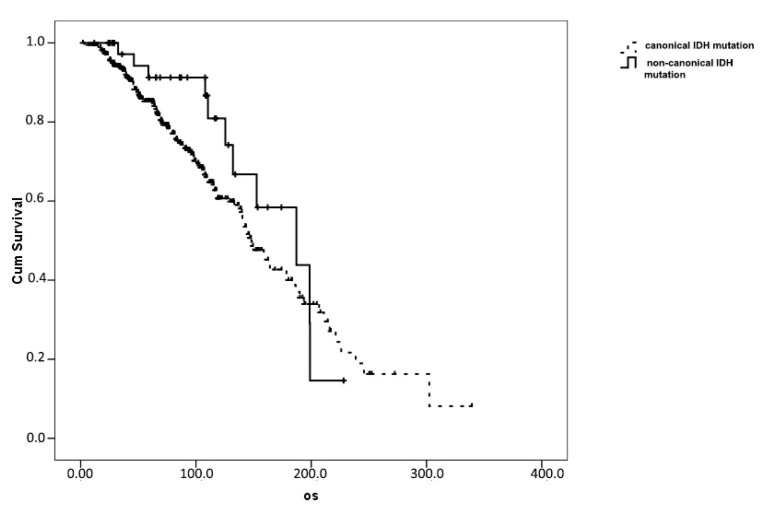
Overall survival comparison in patients with canonical (*n* = 390) and non-canonical (*n* = 43) IDH1 mutations.

No OS difference emerged when comparing patients with R132H IDH1 (mOS 148.1, 95%CI 131.9–164.4 months) and non-canonical IDH1 mutations (187.2, 95% 114.0–260.3 months; *p* = 0.185. Figure 2).

Age, presence/absence of the 1p19q codeletion, and the extension of surgery were all variables affecting the prognosis of patients (Table 3).

A multivariate analysis was performed. Variables included were age, type of IDH1 mutation, extension of primary surgery, and type of treatment received after surgery. In this model, only the extension of surgery (total vs. partial; *p* = 0.001 or resection vs. biopsy; *p* = 0.003), the presence of 1p19q codeletion (*p* = 0.001), and the presence of a non-canonical mutation (*p* = 0.041) were confirmed as variables that were related to improved survival (Table 4).

We supposed that the different percentages of patients harboring the 1p19q codeletion (25.6 vs. 40.2%, in patients with and without a non-canonical mutation, respectively, *p* = 0.017) influenced the overall survival analysis.

Thus, we performed an exploratory survival comparison, only assessing patients without the 1p19q codeletion.

**Figure 3:**
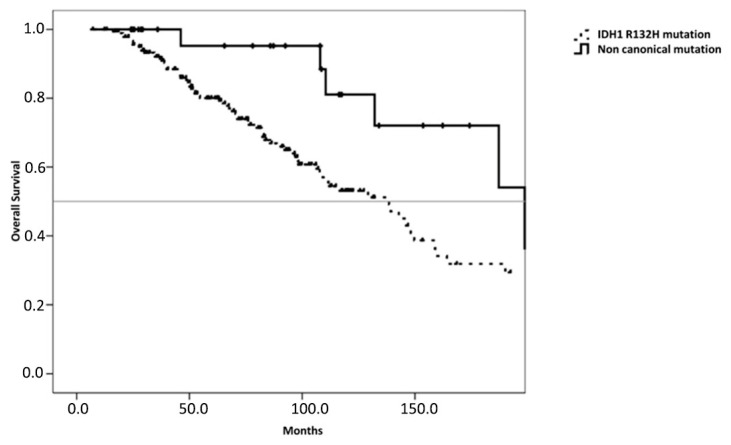
Comparison (log-rank) between patients with canonical (*n* = 187) and non-canonical (*n* = 29) IDH1 mutations in patients without 1p19q codeletion.

In this population, we found a significantly longer survival time for patients with non-canonical IDH1 mutations (198.6, 95% CI 129.7–267.6) compared to those with the R132H IDH1 mutation (138.5, 95%CI 109.7–167.3; *p* = 0.037, see Figure 3).

## 4. Discussion

In the present study, we assessed the clinical impact of non-canonical IDH1 mutation on a population of patients with WHO grade II–III IDH1 mutant gliomas. Notably, we failed to show an OS difference between patients with IDH1 R132H and non-canonical IDH1 mutations. Possible limitations to our findings include the retrospective nature of the study and the small number of patients included who harbored non-canonical mutations, which consequently limited the statistical power of our analysis to 30% in a post-hoc analysis.

Nonetheless, there are some other possible explanations related to our results.

First of all, an imbalance in the number of patients harboring the 1p19q codeletion.

The 1p19q codeletion is a condition associated with a significantly improved survival rate [1,5]. The higher rate of the codeletion reported in patients with the canonical IDH1 mutation could have hidden the prognostic impact of non-canonical alterations in the univariate analysis. When patients with the codeletion were excluded from the analysis, the subgroup of patients with non-canonical mutations showed a longer OS compared to those with the IDH1 R132H mutation. This aspect could also somewhat explain the discrepancy observed in the log-rank (univariate) and multivariate analysis. Indeed, the positive prognostic role observed for non-canonical IDH1 alterations, emerged only in the multivariate assessment when the 1p19q codeletion was also included as an independent variable associated with prognosis.

Previous series strongly suggest that patients with IDH2 mutated glioma more frequently host a 1p19q codeletion than those with IDH1 alterations [17,18]. In our series, we observed that patients with non-canonical mutations—compared to those with the IDH1 R132H mutation—less frequently presented a 1p19q codeletion (25.6 vs. 45.6%, *p* = 0.017).

We decided to exclude patients with IDH2 mutations from the present analysis since tumors with this mutation are related with peculiar biological and clinical features [17,18]. Indeed, the IDH2 mutation appears to be mutually exclusive with PTEN (Phosphatase and tensin homolog), p53, and ATRX mutations [17]. Additionally, the IDH2 mutation is mutually exclusive to IDH1 and could show a different enzymatic and biochemical profile [17,18].

The incidence of non-canonical mutations has been assessed in several studies [2,3,13,14,15,16,21,22,23,24,25]. Hartmann et al. evaluated 1010 patients with WHO grade II–III gliomas, discovering an overall incidence of IDH1 non-canonical mutations of 7.3% [23]. A similar incidence level was detected by Visani et al. [13]. Notably, they reported a possible correlation between non-canonical mutation expression and the grade of the tumor [13].

Poetsch et al. performed a clinical comparison between patients with R132H IDH1 and non-canonical IDH mutant gliomas [25]. This study involved a large series of patients with non-canonical mutations (*n* = 166). These patients more frequently presented multicentric gliomas and tumors localized in the infratentorial region. In addition, patients with non-canonical mutations also frequently had a family history of cancer [25]. In their series, patients harboring a non-canonical IDH1 mutation were more frequently astrocytomas (65.6 vs. 43%), while patients with IDH1 mutations were often oligodendrogliomas (85 vs. 48.3%). There were no significant differences in survival between patients with non-canonical and IDH1 R132H mutant gliomas.

In our study, we did not assess the localization of primary brain tumors and we did not collect data about family cancer history. Similarly to Poetsch et al., we failed to discover an OS advantage for patients with non-canonical mutations in the univariate analysis. However, the presence of non-canonical mutations resulted as a variable associated with prolonged survival in the multivariate comparison. Again, this discrepancy can be partially explained by including the 1p19q codeletion as an independent variable in multivariate comparison. Furthermore, differently from Poetsch et al., we did not include patients with IDH2 mutations, which have been more frequently associated with oligodendroglioma histology. Finally, it should be noted that our analysis, as well as the study by Poetsch, reported a small number of events (32.8 and 15%, respectively) reflecting an immature follow up.

We decided to include the surgical approach as an independent variable associated with survival. To the best of our knowledge, we ignore which could be the best surgical approach. Nonetheless, the available data strongly suggest that maximal safe surgical extension should always be considered, as it could be associated with prolonged survival [26,27]. Thus, assuming that surgical extension and approach could impact survival, we decided to include this variable in the multivariate analysis.

The younger age detected in patients with non-canonical IDH1 mutations is in line with previous studies [13,25].

Each IDH mutation is associated with different enzymatic activity. IDH mutations generally occur in the arginine residue, which is involved in substrate recognition [28,29]. In IDH mutated cells, the production of α-ketoglutarate (which is the usual product of the enzymatic reaction) from isocitrate is shifted to the production of D-2-hydroxyglutarate (D-2-HG) thanks to the neomorphic activity of IDH mutant dimers [28,29]. Both the reduction in α-ketoglutarate (α-KG) and D-2-HG levels mediate carcinogenesis through epigenetic alterations and metabolic/redox intracellular reprogramming [29].

It is possible that the different enzymatic activity of IDH1 could mediate a different outcome in terms of survival. In 2014, Push S et al. demonstrated that D-2-hydroxyglutarate levels increased in the order of R132H-R132C-R132S/R132G/R132L [30]. Similarly, Matteo DA et al. confirmed that common IDH1 mutations have moderate catalytic efficiencies for D-2-hydroxyglutarate production, whereas non-canonical mutations have either very low or very high efficiencies [31].

Finally, we did not assume tumor grade as a distinct variable associated with prognosis in our series. We made this decision due to the conflicting currently contested role of tumor grade on prognosis estimation. Indeed, in our previous report, the tumor grade seemed to affect the prognosis of patients with glioma, while other studies did not confirm this finding [32,33,34,35].

## 5. Conclusions

Our study failed to show a strong correlation between the presence of IDH1 non-canonical mutations and survival in patients with IDH1 mutated grade II/III gliomas. Notably, patients harboring these rare mutations less often presented the 1p19q codeletion and tend to be younger than those with IDH1 canonical alterations. When the survival analysis was restricted to patients without the 1p19q codeletion, non-canonical mutations were associated with improved survival. The results observed in our cohort should be validated in larger series.

## Figures and Tables

**Table 1:** IDH1 mutations detected in the overall population.

IDH1 Mutation	Patients (Percentage)
R132H	390 (90.1%)
R132C	19 (4.4%)
R132G	12 (2.8%)
R132S	9 (2.1%)
R132L	3 (0.7%)

**Table 2:** Patients characteristics and comparison (Fisher test) between IDH1 canonical and IDH1 non-canonical mutation. * Statistically significant variables.

Variables	Overall Population	IDH1 Canonical	IDH1 Non-Canonical	*p*-Value
	(*n* = 433)	(*n* = 390)	(*n* = 43)	
**Median age years (range)**	38 (18–76)	39 (18–76)	29 (19–47)	<0.001 *
**Gender**				0.675
**Male**	253 (58.4%)	229 (58.7%)	24 (55.8%)	
**Female**	180 (41.6%)	161 (41.3%)	19 (44.2%)	
**Grade**				0.675
**2**	269 (62.1%)	239 (61.3%)	30 (69.8%)	
**3**	164 (37.9%)	151 (38.7%)	13 (30.2%)	
**1p19q Codeletion**				0.017 *
**Present**	167 (38.6%)	157 (40.2%)	10 (25.6%)	
**Absent**	216 (49.9%)	187 (48.0%)	29 (67.4%)	
**Miss**	50 (11.5%)	46 (11.8%)	4 (7.0%)	
**Surgical Approach**				0.039 *
**Biopsy**	36 (8.2%)	35 (9.0%)	1 (2.2%)	
**Partial Resection**	254 (58.7%)	233 (59.7%)	21 (48.7%)	
**Total resection**	127 (29.3%)	108 (27.7%)	19 (44.7%)	
**Miss**	16 (3.8%)	14 (3.6%)	2 (4.4%)	
**Post surgical treatment**				0.332
**Follow up**	165 (38.1%)	145 (37.2%)	20 (46.6%)	
**Radiotherapy**	67 (15.5%)	61 (15.6%)	6 (14.1%)	
**Chemotherapy**	40 (9.2%)	39 (10.0%)	1 (2.2%)	
**Radiotherapy and Chemotherapy**	149 (34.4%)	134 (34.3%)	15 (34.9%)	
**Miss**	12 (2.8%)	11 (2.9%)	1 (2.2)	

**Table 3:** Patient characteristics and comparison (Fisher test) between IDH1 canonical and IDH1 non-canonical mutation. * Statistically significant variables.

Variables	Overall Population	IDH1 Canonical	IDH1 Non-Canonical	*p*-Value
	(*n* = 433)	(*n* = 390)	(*n* = 43)	
**Median age years (range)**	38 (18–76)	39 (18–76)	29 (19–47)	<0.001 *
**Gender**				0.675
**Male**	253 (58.4%)	229 (58.7%)	24 (55.8%)	
**Female**	180 (41.6%)	161 (41.3%)	19 (44.2%)	
**Grade**				0.675
**2**	269 (62.1%)	239 (61.3%)	30 (69.8%)	
**3**	164 (37.9%)	151 (38.7%)	13 (30.2%)	
**1p19q Codeletion**				0.017 *
**Present**	167 (38.6%)	157 (40.2%)	10 (25.6%)	
**Absent**	216 (49.9%)	187 (48.0%)	29 (67.4%)	
**Miss**	50 (11.5%)	46 (11.8%)	4 (7.0%)	
**Surgical Approach**				0.039 *
**Biopsy**	36 (8.2%)	35 (9.0%)	1 (2.2%)	
**Partial Resection**	254 (58.7%)	233 (59.7%)	21 (48.7%)	
**Total resection**	127 (29.3%)	108 (27.7%)	19 (44.7%)	
**Miss**	16 (3.8%)	14 (3.6%)	2 (4.4%)	
**Post surgical treatment**				0.332
**Follow up**	165 (38.1%)	145 (37.2%)	20 (46.6%)	
**Radiotherapy**	67 (15.5%)	61 (15.6%)	6 (14.1%)	
**Chemotherapy**	40 (9.2%)	39 (10.0%)	1 (2.2%)	
**Radiotherapy and Chemotherapy**	149 (34.4%)	134 (34.3%)	15 (34.9%)	
**Miss**	12 (2.8%)	11 (2.9%)	1 (2.2)	

**Table 4:** Patient characteristics and comparison (Fisher test) between IDH1 canonical and IDH1 non-canonical mutations. * Statistically significant variables.

Variables	Overall Population	IDH1 Canonical	IDH1 Non-Canonical	*p*-Value
	(*n* = 433)	(*n* = 390)	(*n* = 43)	
**Median age years (range)**	38 (18–76)	39 (18–76)	29 (19–47)	<0.001 *
**Gender**				0.675
**Male**	253 (58.4%)	229 (58.7%)	24 (55.8%)	
**Female**	180 (41.6%)	161 (41.3%)	19 (44.2%)	
**Grade**				0.675
**2**	269 (62.1%)	239 (61.3%)	30 (69.8%)	
**3**	164 (37.9%)	151 (38.7%)	13 (30.2%)	
**1p19q Codeletion**				0.017 *
**Present**	167 (38.6%)	157 (40.2%)	10 (25.6%)	
**Absent**	216 (49.9%)	187 (48.0%)	29 (67.4%)	
**Miss**	50 (11.5%)	46 (11.8%)	4 (7.0%)	
**Surgical Approach**				0.039 *
**Biopsy**	36 (8.2%)	35 (9.0%)	1 (2.2%)	
**Partial Resection**	254 (58.7%)	233 (59.7%)	21 (48.7%)	
**Total resection**	127 (29.3%)	108 (27.7%)	19 (44.7%)	
**Miss**	16 (3.8%)	14 (3.6%)	2 (4.4%)	
**Post surgical treatment**				0.332
**Follow up**	165 (38.1%)	145 (37.2%)	20 (46.6%)	
**Radiotherapy**	67 (15.5%)	61 (15.6%)	6 (14.1%)	
**Chemotherapy**	40 (9.2%)	39 (10.0%)	1 (2.2%)	
**Radiotherapy and Chemotherapy**	149 (34.4%)	134 (34.3%)	15 (34.9%)	
**Miss**	12 (2.8%)	11 (2.9%)	1 (2.2)	

## Data Availability

The datasets used and/or analyzed during the current study are available from the corresponding author on reasonable request.

## References

[1] Louis D.N., Perry A., Reifenberger G., Von Deimling A., Figarella-Branger D., Cavenee W.K., Ohgaki H., Wiestler O.D., Kleihues P., Ellison D.W. (2016). The 2016 World Health Organization Classification of Tumors of the Central Nervous System: A summary. Acta Neuropathol..

[2] Yan H., Parsons D.W., Jin G., McLendon R., Rasheed B.A., Yuan W., Kos I., Batinic-Haberle I., Jones S., Riggins G.J. (2009). IDH1andIDH2Mutations in Gliomas. N. Engl. J. Med..

[3] Sanson M., Marie Y., Paris S., Idbaih A., Laffaire J., Ducray F., El Hallani S., Boisselier B., Mokhtari K., Hoang-Xuan K. (2009). Isocitrate Dehydrogenase 1 Codon 132 Mutation Is an Important Prognostic Biomarker in Gliomas. J. Clin. Oncol..

[4] Brennan C.W., Verhaak R.G.W., McKenna A., Campos B., Noushmehr H., Salama S.R., Zheng S., Chakravarty D., Sanborn J.Z., Berman S.H. (2013). The Somatic Genomic Landscape of Glioblastoma. Cell.

[5] (2015). The Cancer Genome Atlas Research Network Comprehensive, Integrative Genomic Analysis of Diffuse Lower-Grade Gliomas. N. Engl. J. Med..

[6] Hegi M.E., Diserens A.-C., Gorlia T., Hamou M.-F., De Tribolet N., Weller M., Kros J.M., Hainfellner J.A., Mason W., Mariani L. (2005). MGMTGene Silencing and Benefit from Temozolomide in Glioblastoma. N. Engl. J. Med..

[7] Weller M., Stupp R., Reifenberger G., Brandes A.A., Bent M.J.V.D., Wick W., Hegi M.E. (2009). MGMT promoter methylation in malignant gliomas: Ready for personalized medicine?. Nat. Rev. Neurol..

[8] Wesseling P., Bent M.V.D., Perry A. (2015). Oligodendroglioma: Pathology, molecular mechanisms and markers. Acta Neuropathol..

[9] Van den Bent M.J., Dubbink H.J., Sanson M., van der Lee-Haarloo C.R., Hegi M., Jeuken J.W., Ibdaih A., Brandes A.A., Taphoorn M.J.B., Kros J.M. (2009). MGMT promoter methylation is prognostic but not predictive for outcome to adjuvant PCV chemotherapy in anaplastic oligodendroglial tumors: A report from EORTC Brain Tumor Group Study 26951. J. Clin. Oncol..

[10] Brandes A.A., Tosoni A., Cavallo G., Reni M., Franceschi E., Bonaldi L., Bertorelle R., Gardiman M., Ghimenton C., Iuzzolino P. (2006). Correlations Between O6-Methylguanine DNA Methyltransferase Promoter Methylation Status, 1p and 19q Deletions, and Response to Temozolomide in Anaplastic and Recurrent Oligodendroglioma: A Prospective GICNO Study. J. Clin. Oncol..

[11] Mair M.J., Geurts M., Bent M.J.V.D., Berghoff A.S. (2021). A basic review on systemic treatment options in WHO grade II-III gliomas. Cancer Treat. Rev..

[12] Weller M., Bent M.V.D., Preusser M., Le Rhun E., Tonn J.C., Minniti G., Bendszus M., Balana C., Chinot O., Dirven L. (2020). EANO guidelines on the diagnosis and treatment of diffuse gliomas of adulthood. Nat. Rev. Clin. Oncol..

[13] Visani M., Acquaviva G., Marucci G., Paccapelo A., Mura A., Franceschi E., Grifoni D., Pession A., Tallini G., De Biase D. (2017). Non-canonical IDH1 and IDH2 mutations: A clonal and relevant event in an Italian cohort of gliomas classified according to the 2016 World Health Organization (WHO) criteria. J. Neuro Oncol..

[14] Zacher A., Kaulich K., Stepanow S., Wolter M., Köhrer K., Felsberg J., Malzkorn B., Reifenberger G. (2017). Molecular Diagnostics of Gliomas Using Next Generation Sequencing of a Glioma-Tailored Gene Panel. Brain Pathol..

[15] Chen N., Yu T., Gong J., Nie L., Chen X., Zhang M., Xu M., Tan J., Su Z., Zhong J. (2016). IDH1/2 gene hotspot mutations in central nervous system tumours: Analysis of 922 Chinese patients. Pathology.

[16] Camelo-Piragua S., Jansen M., Ganguly A., Kim J.C., Cosper A.K., Dias-Santagata D., Nutt C.L., Iafrate A.J., Louis D.N. (2011). A Sensitive and Specific Diagnostic Panel to Distinguish Diffuse Astrocytoma From Astrocytosis: Chromosome 7 Gain With Mutant Isocitrate Dehydrogenase 1 and p53. J. Neuropathol. Exp. Neurol..

[17] Wang H.-Y., Tang K., Liang T.-Y., Zhang W.-Z., Li J.-Y., Wang W., Hu H.-M., Li M.-Y., Wang H.-Q., He X.-Z. (2016). The comparison of clinical and biological characteristics between IDH1 and IDH2 mutations in gliomas. J. Exp. Clin. Cancer Res..

[18] Shen X., Voets N.L., Larkin S.J., De Pennington N., Plaha P., Stacey R., Mccullagh J.S.O., Schofield C.J., Clare S., Jezzard P. (2019). A Noninvasive Comparison Study between Human Gliomas with IDH1 and IDH2 Mutations by MR Spectroscopy. Metabolites.

[19] De Biase D., Visani M., Baccarini P., Polifemo A.M., Maimone A., Fornelli A., Giuliani A., Zanini N., Fabbri C., Pession A. (2014). Next Generation Sequencing Improves the Accuracy of KRAS Mutation Analysis in Endoscopic Ultrasound Fine Needle Aspiration Pancreatic Lesions. PLoS ONE.

[20] De Biase D., Visani M., Malapelle U., Simonato F., Cesari V., Bellevicine C., Pession A., Troncone G., Fassina A., Tallini G. (2013). Next-Generation Sequencing of Lung Cancer EGFR Exons 18-21 Allows Effective Molecular Diagnosis of Small Routine Samples (Cytology and Biopsy). PLoS ONE.

[21] Balss J., Meyer J., Mueller W., Korshunov A., Hartmann C., von Deimling A. (2008). Analysis of the IDH1 codon 132 mutation in brain tumors. Acta Neuropathol..

[22] Gravendeel L.A., Kloosterhof N.K., Bralten L.B., van Marion R., Dubbink H.J., Dinjens W., Bleeker F.E., Hoogenraad C.C., Michiels E., French P.J. (2010). Segre- gation of non-p.R132H mutations in IDH1 in distinct molecular subtypes of glioma. Hum. Mutat..

[23] Hartmann C., Meyer J., Balss J., Capper D., Mueller W., Christians A., Felsberg J., Wolter M., Mawrin C., von Deimling A. (2009). Type and frequency of IDH1 and IDH2 mutations are related to astrocytic and oligodendroglial differentiation and age: A study of 1010 diffuse gliomas. Acta Neuropathol..

[24] Metellus P., Coulibaly B., Colin C., de Paula A.M., Vasiljevic A., Taieb D., Barlier A., Boisselier B., Mokhtari K., Figarella-Branger D. (2010). Absence of IDH mutation identifies a novel radiologic and molecular subtype of WHO grade II gliomas with dismal prognosis. Acta Neuropathol..

[25] Poetsch L., Network P., Bronnimann C., Loiseau H., Frénel J.S., Siegfried A., Seizeur R., Gauchotte G., Cappellen D., Carpentier C. (2021). Characteristics of IDH-mutant gliomas with non-canonical IDH mutation. J. Neuro Oncol..

[26] Wijnenga M.M.J., French P.J., Dubbink H.J., Dinjens W.N.M., Atmodimedjo P.N., Kros J.M., Smits M., Gahrmann R., Rutten G.-J., Verheul J.B. (2017). The impact of surgery in molecularly defined low-grade glioma: An integrated clinical, radiological, and molecular analysis. Neuro Oncol..

[27] Jakola A.S., Skjulsvik A.J., Myrmel K.S., Sjåvik K., Unsgård G., Torp S.H., Aaberg K., Berg T., Dai H.Y., Johnsen K. (2017). Surgical resection versus watchful waiting in low-grade gliomas. Ann. Oncol..

[28] Dang L., White D.W., Gross S., Bennett B.D., Bittinger M.A., Driggers E.M., Fantin V.R., Jang H.G., Jin S., Keenan M.C. (2009). Cancer-associated IDH1 mutations produce 2-hydroxyglutarate. Nature.

[29] Pusch S., Schweizer L., Beck A.-C., Lehmler J.-M., Weissert S., Balss J., Miller A.K., Von Deimling A. (2014). D-2-Hydroxyglutarate producing neo-enzymatic activity inversely correlates with frequency of the type of isocitrate dehydrogenase 1 mutations found in glioma. Acta Neuropathol. Commun..

[30] Matteo D.A., Grunseth A.J., Gonzalez E.R., Anselmo S.L., Kennedy M.A., Moman P., Scott D.A., Hoang A., Sohl C.D. (2017). Molecular mechanisms of isocitrate dehydrogenase 1 (IDH1) mutations identified in tumors: The role of size and hydrophobicity at residue 132 on catalytic efficiency. J. Biol. Chem..

[31] Han S., Liu Y., Cai S.J., Qian M., Ding J., Larion M., Gilbert M.R., Yang C. (2020). IDH mutation in glioma: Molecular mechanisms and potential therapeutic targets. Br. J. Cancer.

[32] Franceschi E., Tosoni A., Bartolini S., Minichillo S., Mura A., Asioli S., Bartolini D., Gardiman M., Gessi M., Ghimenton C. (2020). Histopathological grading affects survival in patients with IDH-mutant grade II and grade III diffuse gliomas. Eur. J. Cancer.

[33] Pekmezci M., Rice T., Molinaro A.M., Walsh K.M., Decker P.A., Hansen H., Sicotte H., Kollmeyer T.M., McCoy L.S., Sarkar G. (2017). Adult infiltrating gliomas with WHO 2016 integrated diagnosis: Additional prognostic roles of ATRX and TERT. Acta Neuropathol..

[34] Reuss D.E., Sahm F., Schrimpf D., Wiestler B., Capper D., Koelsche C., Schweizer L., Korshunov A., Jones D.T.W., Hovestadt V. (2015). ATRX and IDH1-R132H immunohistochemistry with subsequent copy number analysis and IDH sequencing as a basis for an “integrated” diagnostic approach for adult astrocytoma, oligodendroglioma and glioblastoma. Acta Neuropathol..

[35] Weller M., Weber R.G., Willscher E., Riehmer V., Hentschel B., Kreuz M., Felsberg J., Beyer U., Löffler-Wirth H., Kaulich K. (2015). Molecular classification of diffuse cerebral WHO grade II/III gliomas using genome- and transcriptome-wide profiling improves stratification of prognostically distinct patient groups. Acta Neuropathol..

